# Non-Invasive Diagnostic Imaging in Kaposi Sarcoma Evaluation

**DOI:** 10.3390/diagnostics15131665

**Published:** 2025-06-30

**Authors:** Carmen Cantisani, Antonio Di Guardo, Marco Ardigò, Mariano Suppa, Salvador Gonzalez, Caterina Longo, Alberto Taliano, Emanuele Rovaldi, Elisa Cinotti, Giovanni Pellacani

**Affiliations:** 1 Unit of Dermatology, Department of Clinical Internal, Anesthesiological and Cardiovascular Sciences, “Sapienza” University of Rome, 00185 Rome, Italy; antonio.diguardo@uniroma1.it (A.D.G.); albertotaliano1@gmail.com (A.T.); emanuele.rovaldi@gmail.com (E.R.); giovanni.pellacani@uniroma1.it (G.P.); 2 Dermatology Unit, Humanitas Research Hospital, IRCCS, 20089 Rozzano, Italy; marco.ardigo@hunimed.eu; 3 Department of Dermatology, Hôpital Erasme—Hôpitaux Universitaires de Bruxelles (HUB), 1070 Brussels, Belgium; dr.marianosuppa@gmail.com; 4 Medicine Department, Universidad de Alcalá, 28805 Madrid, Spain; salvagonrod@gmail.com; 5 Skin Cancer Unit, Arcispedale S. Maria Nuova, IRCCS, 42100 Reggio Emilia, Italy; longo.caterina@gmail.com; 6 Dermatology Unit, Department of Medical, Surgical and Neurological Sciences, University of Siena, 53100 Siena, Italy; elisa.cinotti@unisi.it

**Keywords:** dynamic optical coherence tomography, line-field optical coherence tomography, Kaposi sarcoma, herpes virus 8, histopathology

## Abstract

**Background and Clinical Significance:** Kaposi sarcoma (KS) is a rare angio-proliferative mesenchymal tumor that predominantly affects the skin and mucous membranes but may involve lymph nodes and visceral organs. Clinically, it manifests as red-purple-brown papules, nodules, or plaques, either painless or painful, often with disfiguring potential. The diagnosis is traditionally based on clinical and histopathological evaluation, although non-invasive imaging techniques are increasingly used to support diagnosis and treatment monitoring. We report a case of HHV-8-negative Kaposi sarcoma evaluated with multiple non-invasive imaging modalities to highlight their diagnostic utility. **Case Presentation**: An 83-year-old man presented with multiple painful, violaceous papulo-nodular lesions, some ulcerated, on the lateral aspect of his left foot. Dermoscopy revealed the characteristic rainbow pattern. Dynamic Optical Coherence Tomography (D-OCT) allowed real-time visualization of microvascular abnormalities, identifying large serpentine and branching vessels with clearly delineated capsules. Line-field Optical Coherence Tomography (LC-OCT) showed irregular dermal collagen, vascular lacunae, and the presence of spindle cells and slit-like vessels. Histological analysis confirmed the diagnosis of Kaposi sarcoma, revealing a proliferation of spindle-shaped endothelial cells forming angulated vascular spaces, with red blood cell extravasation and a mixed inflammatory infiltrate. **Conclusions:** Non-invasive imaging tools, including dermoscopy, D-OCT, and LC-OCT, have emerged as valuable adjuncts in the diagnosis and monitoring of KS. These techniques enable in vivo assessment of vascular architecture and tissue morphology, enhancing clinical decision-making while reducing the need for immediate biopsy. Dermoscopy reveals polychromatic vascular features, such as the rainbow pattern, while D-OCT and LC-OCT provide high-resolution insights into vascular proliferation, tissue heterogeneity, and cellular morphology. Dermoscopy, dynamic OCT, and LC-OCT represent promising non-invasive diagnostic tools for the assessment of Kaposi sarcoma. These technologies provide detailed morphological and vascular information, enabling earlier diagnosis and more personalized management. While histopathology remains the gold standard, non-invasive imaging offers a valuable complementary approach for diagnosis and follow-up, particularly in complex or atypical presentations. Ongoing research and technological refinement are essential to improve accessibility and clinical applicability.

## 1. Introduction

Kaposi’s sarcoma (KS) is a rare, angio-proliferative neoplasm associated with human herpesvirus 8 (HHV-8) infection [1,2]. HHV-8 plays a critical role in tumorigenesis by inducing endothelial cell proliferation, inflammation, and immune evasion [3]. The virus encodes oncogenic proteins that promote angiogenesis and disrupt cellular homeostasis. KS typically arises in immunocompromised individuals, such as those with HIV/AIDS or organ transplant recipients, due to the loss of immune surveillance against HHV-8-infected cells [4,5]. Although infection with HHV-8 is essential for the development of Kaposi sarcoma, it is not sufficient on its own. The pathogenesis of KS is multifactorial, involving a complex interplay between genetic susceptibility, immune status, and environmental exposures [6,7,8,9]. All clinical variants of KS share a common viral etiology, yet disease expression is shaped by these additional cofactors. The disease is significantly more prevalent in HIV-positive individuals and organ transplant recipients, underscoring the importance of immune competence in controlling HHV-8 [10]. The HIV/AIDS epidemic led to a broader demographic spread of KS, particularly among women and in sub-Saharan Africa, regions previously considered low-risk, reinforcing the role of systemic immunodeficiency as a key trigger [11,12]. In addition to systemic immune dysfunction, local immune alterations—such as those seen in lymphedema—can also favor KS development. The disease progresses through four clinical variants: classic, endemic, iatrogenic, and epidemic (AIDS-related) KS, each with distinct epidemiological and clinical characteristics [13,14,15]. It manifests in various clinical forms, ranging from indolent cutaneous lesions to aggressive systemic diseases. Clinically, KS presents as violaceous macules, plaques, or nodules, often affecting the lower extremities, mucosa, or visceral organs. The progression of KS varies depending on the clinical subtype and host immune status. Classic KS follows an indolent course with slow lesion progression, while epidemic KS in HIV-positive individuals can exhibit rapid dissemination involving the skin, mucosa, and internal organs [13,14]. Iatrogenic KS, seen in immunosuppressed transplant recipients, may regress upon modification of immunosuppressive therapy. Idiopathic KS, though rare, may follow an unpredictable course. Without treatment, KS can progress to extensive cutaneous involvement, lymphedema, and life-threatening visceral disease. Visceral KS occurs when the disease extends beyond the skin and mucous membranes to involve internal organs, most commonly the gastrointestinal (GI) tract, lungs, and liver. It is more frequently seen in HIV-associated and iatrogenic KS due to profound immunosuppression. Symptoms depend on the affected organs—GI involvement may cause bleeding, obstruction, or diarrhea, while pulmonary KS can lead to respiratory distress, hemoptysis, and pleural effusions [16,17,18]. The diagnosis of KS relies on clinical, histopathological, and increasingly, non-invasive imaging techniques. Diagnosis requires endoscopy, bronchoscopy, or imaging techniques such as CT scans. Biopsy confirmation is often necessary. Treatment includes systemic chemotherapy, antiretroviral therapy (in HIV-positive patients), and targeted therapies for symptom management and disease control [17,18]. The management of KS depends on disease extent, immune status, and underlying conditions. Localized cutaneous KS may be treated with cryotherapy, imiquimod, intralesional chemotherapy, or laser therapy. Systemic therapy, including pegylated liposomal doxorubicin and paclitaxel, is indicated for advanced or disseminated disease. In HIV-associated KS, antiretroviral therapy (ART) remains a cornerstone of treatment, often leading to lesion regression [17]. Emerging therapies targeting angiogenesis and immune modulation, such as checkpoint inhibitors and anti-VEGF agents, hold promise for refractory cases. Non-invasive imaging techniques, including dermoscopy, Reflectance Confocal Microscopy (RCM), Dynamic Optical Coherence Tomography (D-OCT), and Line-field Confocal Optical Coherence Tomography (LC-OCT), are increasingly used to aid in the diagnosis and monitoring of Kaposi sarcoma (KS). Dermoscopy has revealed characteristic features such as bluish–reddish coloration, scaly surface, small brown globules, and most notably the polychromatic “rainbow” pattern, which is observed exclusively under polarized light [18,19,20]. This rainbow phenomenon has been linked to optical effects such as diffraction grating and birefringence, potentially influenced by closely packed vascular structures or hyaline globules within nodular lesions. More recent studies have expanded the dermoscopic spectrum, identifying additional features like white lines, white and dot clods, coiled vessels, and the collarette sign, further highlighting the heterogeneity of KS presentation. RCM has shown promise in correlating in vivo imaging with histological findings [21]. Key confocal features include dilated vascular channels in the papillary dermis, extravasated erythrocytes, and linear canalicular structures representing neovascularization. Other characteristic findings include non-edged papillae, inflammatory infiltrates, and net-like dermal fibrillar structures. While RCM demonstrates high sensitivity in detecting KS-specific changes, specificity remains limited, and standardized diagnostic criteria are lacking due to the scarcity of comparative studies. D-OCT offers both cross-sectional and en-face imaging, allowing the visualization of increased vascularity, mesh-like tortuous vessels, and papulo-nodular architecture in untreated lesions [22]. Notably, following treatment (e.g., with topical imiquimod), D-OCT can detect vascular thinning and reduced dermo-epidermal attenuation, supporting its role in therapy monitoring as well as diagnosis [23]. LC-OCT, a newer hybrid modality combining features of RCM and OCT, has shown promise in identifying linear dark spaces in the upper dermis, corresponding to dilated, slit-like vascular channels on histology [24]. These dark spaces often appear irregular and intersecting, accurately matching the tortuous vascular architecture seen in KS tissue sections. In preliminary studies, these findings were present in all examined cases, suggesting a potential role for LC-OCT in in vivo microvascular assessment of KS [25].

We described a case of Kaposi sarcoma evaluated with multiple non-invasive diagnostic imaging, enabling a comprehensive assessment of both vascular and structural changes characteristic of KS.

## 2. Case Presentation

**Figure 1:**
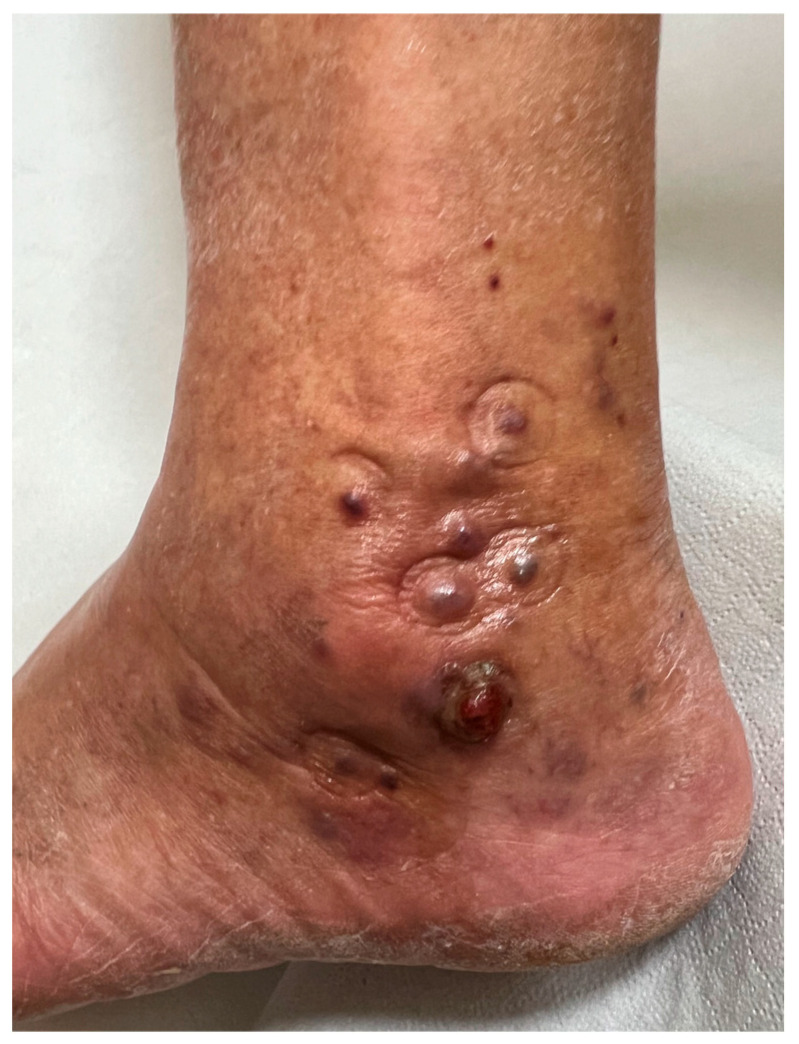
Clinical aspect with multiple red–violet painful papulo-nodular lesions, the largest ulcerated on the external malleolus of the left foot.

**Figure 2:**
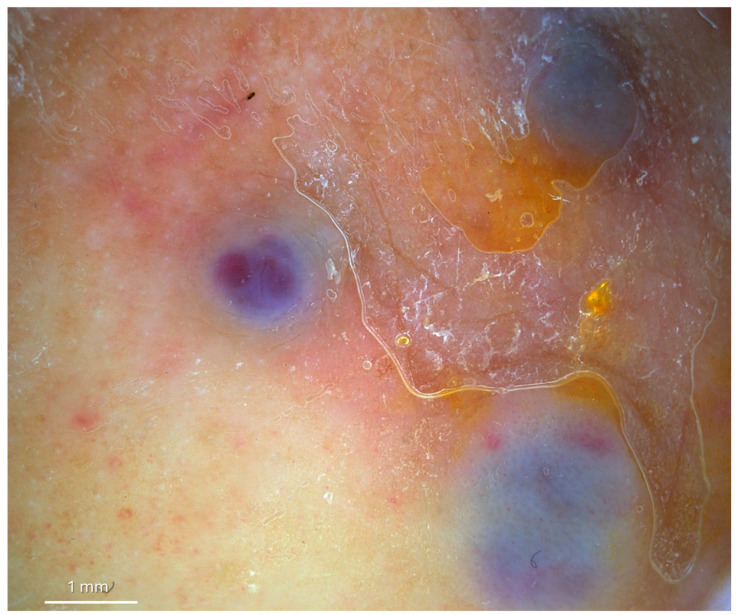
Dermoscopy showed a rainbow pattern of white lines or dotted vessels (10× magnification).

**Figure 3:**
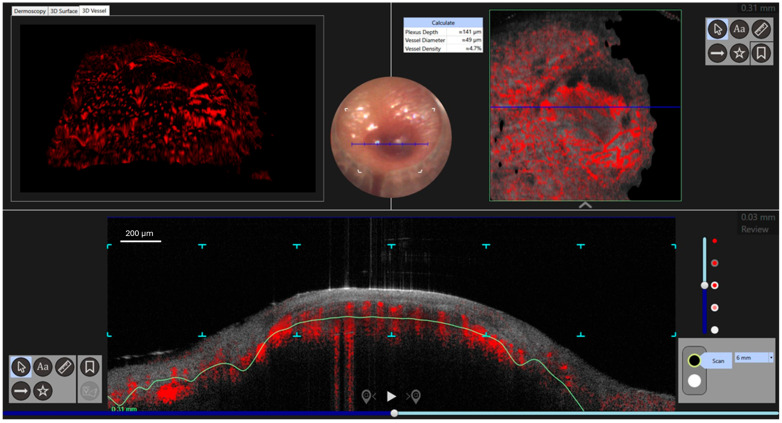
Dynamic OCT showed mottle, serpentine vessels with a plexus depth of 141 μm, vessel diameter of 49 μm, vessel density of 4.7%, an en face vascular branching pattern on the transversal image, and a well-defined nodule of atypical vessels with slit-like vessels.

**Figure 4:**
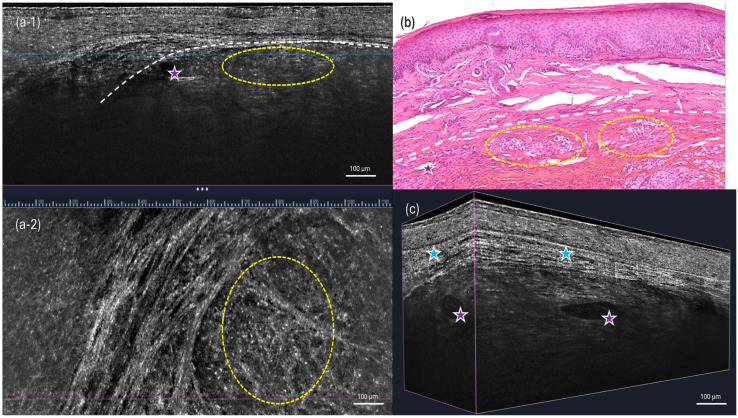
LC-OCT images and histology. (**a-1**,**a-2**) Vertical and horizontal (en face) views of the 3D volumetric LC-OCT image. (**b**) Three-dimensional LC-OCT reconstructions of the lesion. (**c**) Histological section of the corresponding lesion, stained with hematoxylin and eosin (H&E, 20×). The nodule apparent on H&E correlates with nodular structures seen on vertical LC-OCT images (white dashes). Elongated hypo-reflective dark areas of larger diameter showing floating hyper-reflective oval structures in the tumor nodule (purple stars), corresponding to vascular lacunae. Irregular thin hypo-reflective dark areas, corresponding to slit-like vessels (blue stars), can be seen above the nodule and between tumor cells (yellow dashes).

An 83-year-old male presented with multiple painful, red–violet, papulo-nodular lesions, some of which were ulcerated, located on the external malleolus of the left foot (Figure 1). Dermoscopic, D-OCT, and LC-OCT images were acquired on multiple different lesions. D-OCT images were taken using the VivoSight^®^ device (Michelson Diagnostics, Weavering, UK). D-OCT is a non-invasive imaging device that allows for the visualization of vessel morphology thanks to its dynamic component. This imaging technique has a penetration depth of up to 1.5 mm and an axial resolution of 3–15 µm. LC-OCT images were acquired with the deepLive™ system (DAMAE Medical, Paris, France). LC-OCT offers three different modalities of in vivo imaging: Vertical slice images, Horizontal en face images, and three-dimensional reconstructions of the skin. It allows the visualization of cytologic structures of the epidermis and dermis, provided by both vertical histology-like images and en face horizontal images. With high axial and lateral resolutions (1.1 µm and 1.3 µm) and a penetration depth of up to 500 µm, this technology proves to be particularly relevant for diagnosis as well as treatment monitoring of skin tumors. Multiple vertical videos covering the different lesions were acquired, as well as 3D scans on a field of view of 1.2 mm × 0.5 mm × 0.5 mm. Each LC-OCT 3D scan took approximately 20 s, during which the operator of the probe ensured decent stability. In the case that the probe moved slightly during the acquisition, “Motion Freeze” algorithms are instantly applied to correct the detected movement and avoid artifacts. Dermoscopy revealed a rainbow pattern, while Dynamic Optical Coherence Tomography (D-OCT) provided real-time, high-resolution visualization of tissue microstructures and abnormal vascular networks. This enabled the assessment of abnormal vasculature, showing large serpentine vessels and a branching pattern on the en face section, which was clearly identifiable even on the transversal section, along with a well-defined capsule (Figure 2 and Figure 3). More specifically, D-OCT showed a plexus depth of 141 μm, a vessel diameter of 49 μm, and a vessel density of 4.7%. LC-OCT demonstrated features such as irregular dermal collagen structures, tissue heterogeneity, and the presence of vascular lacunae (Figure 4). Elongated, hypo-reflective dark spaces of larger diameter containing floating, hyper-reflective oval structures—corresponding to vascular lacunae—are visible within the tumor nodule. In addition, irregular, thin hypo-reflective dark areas, indicative of slit-like vessels, are observed above the nodule and interspersed between tumor cells (Figure 4). Criteria such as slit-like vessels and elongated spindle cells were also visible in the epidermis. Histopathological examination confirmed the diagnosis of Kaposi sarcoma, revealing vascular proliferation composed of spindle-shaped endothelial cells forming irregular, angulated spaces reminiscent of vascular slits (Figure 4). The lesion also showed areas of red blood cell extravasation and a mixed inflammatory infiltrate, consistent with the typical microscopic features of this neoplasm.

## 3. Discussion

Non-invasive diagnostic tools have increasingly gained attention in the early detection and monitoring of KS, offering valuable alternatives to traditional histopathological examination [21,23,25]. Dermoscopy, high-frequency ultrasound, and advanced optical imaging techniques such as confocal microscopy and dynamic OCT have shown promise in enhancing diagnostic accuracy. These modalities enable the visualization of characteristic vascular patterns, structural alterations, and tissue changes, potentially reducing the need for invasive biopsies. By integrating these non-invasive approaches, clinicians can improve early diagnosis, optimize patient management, and refine therapeutic strategies. Dermoscopy reveals characteristic vascular patterns, including linear and curved vessels, and a rainbow pattern. Dermoscopy has emerged as a valuable non-invasive tool in the evaluation of KS lesions [26]. Characteristic dermoscopic features of KS include the presence of a multicolored pattern, with varying shades of red, purple, and brown, as well as the detection of specific vascular structures such as linear and curved vessels [19,20]. These features, although not specific, will aid in addressing suspicion and differentiating KS from other vascular and pigmented lesions, contributing to early and accurate diagnosis [27]. High-resolution imaging techniques, such as Dynamic Optical Coherence Tomography (OCT) and Line-field Confocal OCT (LC-OCT), enhance the diagnostic process by providing real-time visualization of vascular proliferation and tissue architecture [22]. Dynamic Optical Coherence Tomography (D-OCT) provides real-time, high-resolution visualization of tissue microstructures and vascular networks. In KS, dynamic OCT enables the assessment of abnormal vasculature, helping to distinguish it from benign vascular proliferations [23]. Although not widely available, this technology offers promising applications in the early detection and monitoring of KS. Big serpentine vessels, branching patterns on the en face section, are well visible even on the transversal section, with a definite capsule substituted by fibrosis after treatment. However, insufficient resolution is available to detect cytologic structures and differentiate different cell types present in the lesion. Reflectance Confocal Microscopy shows inflammatory cells in epidermis and dermis (single or in aggregates), spindle cells, stroma, anastomosing, newborn, increased numbers, dilated vessels, extravasated erythrocytes, and deposits of hemosiderin [21]. Line-field Confocal Optical Coherence Tomography (LC-OCT) combines the benefits of OCT with confocal microscopy, offering cellular-level resolution of skin lesions. This method is particularly useful, as in our case of KS diagnosis, as it provides detailed cross-sectional imaging that allows for architectural assessment comparable to histopathology. It can reveal features such as vascular proliferation, irregular dermal collagen structures, and tissue heterogeneity, enhancing the diagnostic accuracy of KS without requiring invasive biopsy. [25,28] Furthermore, a complete examination of a lesion with LC-OCT comprising multiple video acquisitions and multiple 3D images takes approximately 2 to 3 min, which demonstrates the relevancy of this method in clinical practice for diagnostic purposes. LC-OCT visualization of the internal structures of the Kaposi nodes remains limited to 500 µm. Histopathological examination remains the gold standard, showing spindle-shaped endothelial cells, slit-like vascular spaces, and hemosiderin deposition [29].

The growing availability of non-invasive imaging tools in dermatology has expanded the possibilities for improving diagnostic accuracy in complex skin conditions, including Kaposi sarcoma. Techniques such as dermoscopy, Reflectance Confocal Microscopy (RCM), Optical Coherence Tomography (OCT), and LC-OCT offer the potential to enhance clinical assessment by providing in vivo, high-resolution visualization of morphological and vascular features. These tools can support a more targeted diagnostic approach, assist in selecting the most appropriate biopsy site, and contribute to monitoring disease evolution or treatment response over time [30]. Their integration into clinical workflows may lead to earlier intervention, better treatment planning, and more personalized follow-up strategies. However, despite their promising role, the widespread adoption of these technologies in daily practice is still hindered by several limitations. Access to high-end imaging devices remains limited in many healthcare settings due to economic constraints and uneven territorial distribution. Moreover, the interpretation of these advanced images requires specific training and experience, which may not be routinely available across all dermatology units. Additional barriers include the current lack of standardized diagnostic criteria for Kaposi sarcoma in non-invasive imaging and the potential risk of misinterpretation in the absence of histopathological confirmation. While these technologies represent a valuable complement to traditional diagnostics, further efforts are needed to promote their accessibility, refine their diagnostic algorithms, and validate their clinical utility in broader real-life settings.

## 4. Conclusions

Non-invasive imaging techniques such as dermoscopy, dynamic OCT, and LC-OCT represent significant advancements in the diagnosis and management of KS. By providing detailed morphological and vascular information, these modalities contribute to early detection, reducing the need for invasive procedures and improving patient outcomes. They also allow for easier and more detailed treatment follow-up, as morphological and cytologic changes can be closely monitored with non-invasive imagery. This article breaks new ground by describing both methodologies side by side, shedding light on their distinct aspects and providing valuable additional insights. Further research and technological advancements may enhance their diagnostic capabilities and expand their clinical applications.

## Data Availability

The original contributions presented in this study are included in the article. Further inquiries can be directed to the corresponding author.

## Abbreviations

The following abbreviations are used in this manuscript:

KS	Kaposi Sarcoma
HHV-8	Herpesvirus 8
d-OCT	Dynamic Optical Coherence Tomography
Lc-OCT	Line-field Optical Coherence Tomography
